# Protocol to investigate replication kinetics of Kaposi’s sarcoma-associated herpesvirus using single-molecule analysis of replicated DNA

**DOI:** 10.1016/j.xpro.2025.104287

**Published:** 2025-12-22

**Authors:** Rajnish Kumar Singh, Dipayan Bose, Erle S. Robertson

**Affiliations:** 1 Department of Otorhinolaryngology, University of Pennsylvania, Philadelphia, PA 19104, USA

**Keywords:** Cancer, Microbiology, Molecular Biology

## Abstract

Single-molecule analysis of replicated DNA (SMARD) is a powerful tool to study DNA replication by visualizing *de novo*-incorporated nucleotide analogs in individual DNA molecules. Here, we present the protocol to investigate replication kinetics of Kaposi’s sarcoma-associated herpesvirus using SMARD. We describe steps for seeding cells, nucleotide analog pulsing of cells, preparation and digestion of agarose plugs followed by pulsed-field gel electrophoresis, probe labeling, DNA stretching, and fluorescence imaging to map replication origins and fork progression.

For complete details on the use and execution of this protocol, please refer to Verma et al.^1^ and Singh et al.^2^

## Highlights

• Steps for pulsing cells with sequential nucleotide analogs for replication labeling
• Procedures for embedding cells in agarose plugs and isolating high-molecular-weight DNA
• Instructions for linearizing KSHV DNA using restriction digestion and PFGE
• Guidance on stretching viral DNA and detecting IdU and CldU by fluorescence microscopy

## Abstract (editor highlights)

Publisher’s note: Undertaking any experimental protocol requires adherence to local institutional guidelines for laboratory safety and ethics.

## Graphical abstract

**Figure:**
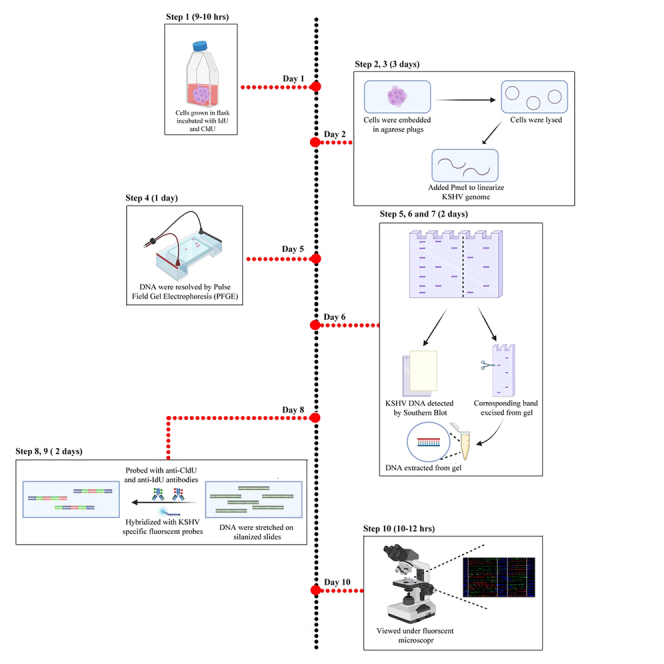

## Teaser

Single-molecule analysis of replicated DNA (SMARD) is a powerful tool to study DNA replication by visualizing *de novo*-incorporated nucleotide analogs in individual DNA molecules. Here, we present the protocol to investigate replication kinetics of Kaposi’s sarcoma-associated herpesvirus using SMARD. We describe steps for seeding cells, nucleotide analog pulsing of cells, preparation and digestion of agarose plugs followed by pulse field gel electrophoresis, probe labeling, DNA stretching, and fluorescence imaging to map replication origins and fork progression.

## Before you begin

Single molecule analysis of the replicated DNA (SMARD) is a technique that precisely identifies regions undergoing replication on individual DNA molecules. This method can be applied to study the replication dynamics of cellular genomes, or of pathogens including bacteria and viruses (For example, Kaposi’s sarcoma associated herpesvirus, KSHV).^1^^,^^3^^,^^4^^,^^5^

In this protocol, we provide a step-by-step description of an adaptation of the original SMARD technique that enables detection of replication initiation sites on individual KSHV genomes using fluorescence microscopy. This version is optimized for episomal KSHV genomes maintained in latently infected cells; analysis of integrated viral genomes (e.g., HPV) would require additional considerations such as mapping of integration junctions, restriction-site availability, and potential sequence deletions. The approach can be further modified for use with other integrated viruses or pathogens.

SMARD has been used to define where replication initiates, the direction and progression of replication forks, and the influence of regulatory or environmental factors on replication dynamics.^6^^,^^7^^,^^8^ To analyze a specific genomic region, locus-specific probes are used for orientation and detection by immunofluorescence. Here, we examined replication origin activity and fork progression on the KSHV genome using the KSHV-positive BC3 cell line, which maintains 80–100 latent viral genome copies as chromatinized episomes.^2^^,^^9^^,^^10^

Sequential labeling with halogenated nucleotides 5′-iodo-2′-deoxyuridine (IdU) and 5′-chloro-2′-deoxyuridine (CldU) enables visualization of replication fork direction and abundance at single-molecule resolution. KSHV DNA molecules are distinguished from host DNA by hybridization with biotinylated, KSHV-specific probes detected by fluorescence in situ hybridization.

The labeled KSHV double-stranded DNA molecules incorporate nucleotide analogs throughout their length, allowing visualization over background fluorescence. The FISH-based identification of viral DNA ensures distinction from cellular DNA, though some proportion of viral genomes may remain unlabeled and appear as faint or “ghost” molecules. Together, these optimizations provide a robust framework for mapping replication origins, fork progression, and termination zones in viral genomes and highlight potential limitations that users should consider when applying SMARD to low-copy episomal DNA.

### Innovation

This protocol presents an optimized application of the Single Molecule Analysis of Replicated DNA (SMARD) technique for investigating the replication dynamics of Kaposi’s Sarcoma–associated Herpesvirus (KSHV). While SMARD has been previously used to study chromosomal replication, its adaptation to a viral episome represents a significant methodological advancement. This version details every step necessary to isolate, label, and visualize KSHV episomal DNA at single-molecule resolution, enabling researchers to study viral replication kinetics within the host cellular environment. The innovation lies in optimizing labeling, purification, and fluorescence detection steps for low-copy viral episomes that coexist with host DNA, ensuring signal specificity and minimal background. In addition, the protocol introduces refined troubleshooting notes to address challenges associated with viral genome heterogeneity, replication origin identification, and DNA molecule alignment. By combining these optimizations with detailed procedural guidance and quantification parameters, this protocol provides a reproducible framework for analyzing replication fork progression and origin usage in episomal viruses. The approach can be readily adapted to other latent or low-abundance viral genomes and thus expands the applicability of SMARD beyond host genomic replication studies.

### Preparation one

**Timing: 14–16 h**

1. Silanized slides.
  a. Soak slides in 1% SDS solution in a tray for at least 30 minutes.
  b. Take out the slides with the help of a forceps and wash with warm water (45°C–50°C) followed by washing with distilled water.
  c. Soak the slides in HNO_3_: HCl solution (2:1) for 12 hours in a glass tray tightly sealed with saran wrap. Put the glass tray with soaked slides in a fume hood.
  d. After 12 hours of soaking, remove the slides with the help of forceps and rinse thoroughly with distilled water (3–4 times, 3–4 minutes each).
  e. Rinse the slides with methanol in a Coplin jar by moving up and down several times.
  f. Soak the slides in 4% Amino Silane in methanol for 1 hour at 20°C–25°C, with mild shaking.
  g. Rinse the slides in methanol followed by three washes in distilled water (3minutes each).
  h. Rinse the slides with 95% ethanol, air dry and put in desiccator under airtight conditions. It is important to use the slides after 12 to 24 hours of desiccation. Further, the slides loses their stickiness for DNA after long storage (generally 96 hours onwards).

### Preparation two

**Timing: 0.5–1 h**

2. Nucleotide analog (IdU and CldU) stock solution.
  a. 10 mM CldU: Dissolve 128.4 mg of CldU in 48.9 mL H_2_O. Keep this stock solution frozen at −20°C.
  b. 10 mM IdU: Dissolve 205.5 mg IdU in 58.05 mL H_2_O; add 200 μL 10 N NaOH. Keep this stock solution frozen at −20°C.

### Preparation three

**Timing: 0.5–1 h**

3. 10% Sarcosine.
  a. Dissolve 100g of L-Sarcosine in 850 mL of H_2_O.
  b. Make up the volume up to 1 Liter with H_2_O.

### Preparation four

**Timing: 0.5–1 h**

4. 0.5 M EDTA pH 8.0.
  a. Dissolve 186.5g of EDTA in 800 mL H_2_O.
  b. Add 10N NaOH until the solution reaches pH 8.0 and then make up the volume to one liter.

### Preparation five

**Timing: 0.5–1 h**

5. InCert Agarose.
  a. For making plugs: Prepare 1.4% of InCert agarose in Phosphate Buffered Saline (PBS).
  b. Aliquot in 1 mL quantities in 1.5 mL micro-centrifuge tubes and store aliquots at 4°C.

### Preparation six

**Timing: 0.5–1 h**

6. Low melting agarose.
  a. Prepare 0.7% Low melting agarose in Tris-acetate EDTA buffer or Tris-Borate EDTA buffer pH8.0.

### Preparation seven

**Timing: 0.5–1 h**

7. Storage buffer for agarose plugs (50 mM EDTA+ 10 mM Tris-HCl, pH 8).
  a. For 1 Liter: Add 100 mL of 0.5M EDTA and 10 mL of 1M Tris-HCl pH 8.0 to the 890 mL of autoclaved distilled water.

### Preparation eight

**Timing: 0.5–1 h**

8. Pre-digestion buffer for agarose plugs (10mM MgCl_2_ + 10 mM Tris-HCl 8.0).
  a. For 1 Liter: Add 10 mL of 1M MgCl_2_ and 10 mL of 1M Tris-HCl pH 8.0 to the 980 mL of autoclaved distilled water.

## Key resources table

**Table:** 

REAGENT or RESOURCE	SOURCE	IDENTIFIER
**Chemicals, peptides, and recombinant proteins**		

5-Chloro-2′-deoxyuridine	MilliporeSigma	C6891
5-Iodo-2′-deoxyuridine	MilliporeSigma	I7125
Ethylenediaminetetraacetic acid disodium salt dihydrate (EDTA)	MilliporeSigma	E4884
Sarcosine	MilliporeSigma	131776
Phenylmethylsulfonyl fluoride (PMSF)	MilliporeSigma	10837091001
Tris (Hydroxymethyl) Aminomethane	Research Products International	T60040
Glycine	Research Products International	G36050
Sodium Chloride	Research Products International	S23020
Sodium Phosphate, Monobasic	Research Products International	S23185
Sodium Hydroxide Pellets	Research Products International	S24000
Formamide	MilliporeSigma	F7503
Dextran sulfate	MilliporeSigma	D8906
Glutaraldehyde Solution	MilliporeSigma	G7776
Aminosilane	MilliporeSigma	440140
2-Mercaptoethanol	MilliporeSigma	M6250
Biotin-14-dATP	Thermo Fisher Scientific	19524016
YOYO-1 Iodide (491/509)	Thermo Fisher Scientific	Y3601
InCert Agarose	Lonza	50121
Agarose GPG/LMP	American Bio	AB00981
Lambda PFG Ladder	New England Biolabs	N0341S
Yeast Chromosome PFG Marker	New England Biolabs	N0345S
PmeI	New England Biolabs	R0560
Beta Agarase I	New England Biolabs	M0392
Not I restriction enzyme	New England Biolabs	R3189
BamHI restriction enzyme	New England Biolabs	R3136
Proteinase K	Thermo Fisher Scientific	AM2542

**Antibodies**		

Mouse-anti-IdU anti-BrdU (Bromo deoxyuridine) (used 10 times diluted)	BD Fisher Scientific	BDB347580
Rat-anti-CldU anti-BrdU mab (used 10 times diluted)	Accurate Chemical and Scientific Corporation	OBT0030S
Avidin, NeutrAvidin, Alexa Fluor 350 conjugate (used 20 times diluted)	Thermo Fisher Scientific	A11236
Goat anti-Mouse IgG Alexa Fluor 488 (used 10 times diluted)	Thermo Fisher Scientific	A-11001
Goat anti-Rat IgG Alexa Fluor 594 (used 10 times diluted)	Thermo Fisher Scientific	A-11007

**Software and algorithms**		

Fiji (ImageJ) (v.2.14.0)	Rueden et al.^11^	https://imagej.net/ij/

**Other**		

Amarsham Hybond XL	GE Healthcare	50174589
BioNick DNA Labeling System	Thermo Fisher Scientific	18247015

## Step-by-step method details

### Day 1: Seeding the cells

**Timing: 0.5–1 h**

Here we describe steps for growing cells in exponential phase which is required for pulsing step on the following day.

1. Count and seed BC3 cells.
  a. Count exponentially growing body cavity-based lymphoma cells (BC3 cells) using a hemocytometer or an automated cell counter.
  b. Seed cells in a T75 flask at a density of **0.4–0.5 × 10**^**6**^ **cells/mL** in complete RPMI medium containing **10% bovine growth serum (BGS).**
  c. Grow a 50 mL culture for each experimental set.
  d. Place an equal volume of complete RPMI medium in a fresh T75 flask and incubate it alongside the culture flask overnight. This ensures temperature equilibration of the medium for use on the next day.

***Note:*** This protocol can be applied to any suspension or adherent cell line. Adjust seeding density and growth conditions according to the cell type used.

### Day 2 to day 4: Nucleotide analog pulsing of cells, preparation of cell-embedded agarose plugs, and proteinase K digestion

**Timing: 10–12 h**

Here we describe steps for nucleotide pulsing of the cells followed by preparation of agarose plugs embedded with nucleotide analog pulsed cells. The proteinase K digestion of cells embedded in agarose plugs ensure integrity of DNA without shearing.

2. Cell harvesting, growing in nucleotide analog containing cell culture medium and preparation of cells embedded agarose plugs.
  a. CldU Pulsing:
    i. Harvest the cells by centrifugation at 500 x g for 5 minutes.
    ii. Resuspend the cells evenly in 10 mL of cell culture medium and add 125 μL of prepared CldU solution.
    iii. Top the volume to 50 mL using cell culture medium and transfer the whole content in a T75 flask.
    iv. Incubate the culture in a humidified 5% CO_2_ incubator for 4 h.
  b. IdU Pulsing:
    i. Pellet down the cells from “step a” by centrifugation at 500 x g for 5 minutes.
    ii. Resuspend cells evenly in 10 mL of cell culture medium and add 125 μL of prepared IdU solution.
    iii. Top the volume to 50 mL using cell culture medium and transfer the contents to a new T75 flask.
    iv. Incubate the culture in a humidified 5% CO_2_ incubator for 4 h.
  c. Preparation of embedded agarose cell plugs: Harvest the cells from “step b” by centrifugation at 500 x g for 5 minutes.
    i. Wash the cells by resuspending in ice cold 1X PBS followed by centrifugation at 500 x g for 5 minutes.
    ii. Resuspend the cells in ice cold 1X PBS at a concentration of 3x10^7^ cells/mL and place the cell suspension on ice.
    iii. Melt 1 mL of 0.7% InCert agarose (prepared in 1X PBS) by placing on a hot plate tube stand for 4–5 minutes. Alternatively, the agarose can be melted by putting in a water bath.
    iv. Equilibrate cell suspension as well as InCert agarose at 42°C for 5 minutes. Add the InCert agarose to the tube containing cell suspension in equal proportions and mix uniformly by pipetting up and down gently several times. Mixing of cell suspension and agarose results in density of 1.5x10^7^ cells/mL of suspension. High density of cells may result in partial proteinase K and Pme1 digestion. Further, low density of cells can lead to less populated KSHV DNA in the last steps of SMARD.
    v. Using a cut yellow tip (200 μL), dispense the cells-InCert agarose mix into the wells of the plug caster (provided with the CHEF DRII pulse field instrument). Approximately 100 μL of suspension is required for making one plug. Make plugs from the entire suspension as processed plugs can be stored in storage buffer at 4°C for several months. Allow the plugs to solidify by putting the plug caster containing the cells-InCert agarose mix in the cold room/4°C refrigerator for 30–60 minutes.
3. Proteinase K digestion of cells embedded agarose plugs.
  a. Take 25 mL of plug digestion buffer (1% Sarcosine in 0.5M EDTA) in a 50 mL tube.
  b. Using a cut 0.1–10 μL sterile tip, push down the agarose plugs in the digestion buffer. Several plugs can be digested in one tube.
  c. Add 0.25 mL Proteinase K solution (20 mg/mL)/25 mL of plug digestion buffer.
  d. Place the tube at 50°C water bath.
4. Change the plug digestion buffer next day and add fresh Proteinase K. Leave at 50°C water bath for 24 hours.
5. Change the plug digestion buffer again and add fresh Proteinase K. Leave at 50°C water bath for another 24 hours.

### Day 5: Washing the plugs after proteinase K digestion

**Timing: 48–72 h**

Here we describe steps for washing of cell embedded agarose plugs after proteinase K digestion. A proper washing is needed for efficient restriction digestion in the subsequent steps.

6. Rinse the plugs in Tris-EDTA pH8 (TE8) buffer several times in a Petri dish. The used buffer should be removed carefully using a vacuum aspirator or pipetman by tilting the petri-dish. Avoid completely discarding the used buffer to prevent damage to the plugs. Transfer the plugs in a 50 mL tube by pouring slowly through the side of the tube and fill the tube with TE8. Let the plugs wash for one hour at 20°C–25°C with mild shaking.
7. Replace the TE8 washing buffer with fresh TE8 buffer and allow the plug to wash for another 1 hour at 50°C.
8. Replace the TE8 washing buffer with fresh TE8 buffer, and add 100 μL of 100 mM Phenylmethylsulphonyl fluoride (PMSF) and place the tube at 50°C for 30 minutes.
9. Repeat step 3 with fresh TE8 buffer and PMSF for another 30 minutes.
10. Wash the plugs in TE8 buffer at 20°C–25°C for 1 hour with mild shaking.
11. Repeat step 5 with fresh TE8 buffer for another 1 hour.
12. Remove the buffer and put the plugs in fresh TE8 buffer and place at 4°C for 12–24 hours.
13. Transfer the plugs to storage buffer (50 mM EDTA+10 mM Tris-HCl pH 8.0) and leave at 4°C for 12–24 hours. After this initial storage of 12–24 hours, the plugs are ready to use for next step of the protocol.
14. Change the storage buffer every day and keep the plugs at 4°C. The plugs can be stored in the storage buffer for several months at 4°C. This step can serve as pause point before proceeding to the next step of the protocol.

### Day 7: Pme1 digestion of agarose plugs to linearize the KSHV genome

**Timing: 24 h**

Here we describe steps for Pme1 restriction digestion of proteinase K digested agarose plugs. Pme1 cut KSHV episome at single site to linearize the KSHV genome.

15. Rinse the agarose plugs with sterile H_2_O.
16. Rinse the agarose plugs with pre-digestion buffer (10 mM MgCl_2_ + 10 mM Tris-HCl 8.0).
17. Equilibrate the agarose plugs in pre-digestion buffer for at least one hour.
18. Prepare the enzyme mix and set up the Pme1 digestion of plugs. For each plug, make 200 μL of enzyme mix as shown below.

**Table:** 

	For one plug
10 X rCutsmart Buffer	20 μL (1X Final concentration)
Pme1	2 μL or 20U (1U/10 mL of solution)
DdH_2_O	178 μL
Total	200 μL

19. Set up digestion of individual plugs in a separate sterile microcentrifuge. Pipette 200 μL of enzyme mix in each tube as per the number of plugs planned for digestion.
20. Using a cut tip, slowly slide one plug into each of the microcentrifuge tube and put the tubes at 37°C for restriction digestion (12-18 hours).

### Day 8: Pulsed-field gel electrophoresis setup and run

**Timing: 30–36 h**

Here we describe steps for running the pulse field electrophoresis gel to resolve the Pme1 digested DNA.

21. Take agarose plugs from the microcentrifuge tube to a petri dish and wash the plugs with TE8 for 1 hour at 20°C–25°C on a rotor shaker with mild shaking. The plugs can be washed for several hours at 4°C.
22. Dissolve 1.05g low melting agarose in 0.5X TBE (Tris-Borate-EDTA) or 1X TAE (Tris-Acetate-EDTA) to get a 0.7% low melting agarose (LMP). Let the solution cool at 20°C–25°C for 20–30 minutes. Before casting the agarose gel, the plugs were set at the edge of the comb. Place the comb supporting the plugs at the proper place in the casting tray. Slowly pour the agarose in the casting tray near one of the far corners. Special care should be taken so that the plugs stay attached with the combs and don’t fall off. It is always recommended to perform the casting of agarose gel in a cold room, which allows better polymerization of the gel.
23. Take out the comb from the tray and fill the created empty space with agarose.
24. Place the gel in the running tank of the CHEF-DRII PFGE running system. The optimal volume of buffer in the tank is approximately 3 liters. Run PFGE for 32-36 hours. The settings for standard run are: Initial swing= 9, Final swing=14, V/Cm=6.

### Day 9: Transfer of DNA onto a nylon membrane and Southern blot for identification of gel location containing KSHV DNA

**Timing: 24–36 h**

**Figure 1:**
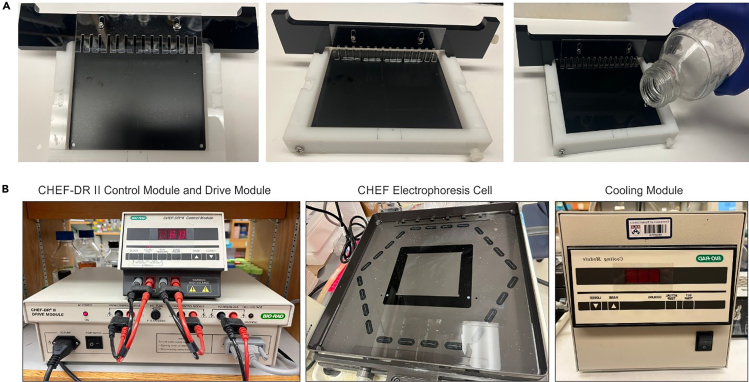
Representative image for gel casting with plugs and instruments used for pulsed-field gel electrophoresis (PFGE) (A) Placing the agarose plugs on comb and casting the gel. (B) CHEF-DRII Control Module and Drive Module (Left), CHEF Electrophoresis Cell (Middle) and Cooling Module (Right).

Here we describe steps for Southern blot transfer of DNA from the pulse field electrophoresis gel to nylon membrane. This allows identification of possible location of KSHV genome agarose gel. A representative figure for PFGE, setting up transfer of DNA and Southern blot is provided in Figure 1.

The composition of required solutions is given below.

**Depurination solution:** 0.25M HCl (Add 20.8 mL of 37% HCl in one-liter ddH_2_O).

**Denaturing solution:** 0.5M NaOH + 1.5M NaCl (20g NaOH + 87.5g NaCl per liter of ddH_2_0).

**Neutralizing Solution:** 1M Tris pH 7.5+1.5 M NaCl (121.1g Tris+ 87.5g NaCl).

**Table:** 20X SSPE (Saline-sodium Phosphate-EDTA) Solution: 1 Liter

		Final concentration
NaCl	175.2g	3 M
NaH2PO4	27.6g	0.23 M
EDTA	7.4g	0.025 M
NaOH	8.5g	0.2125 M

**Table:** 20X SSC Buffer (Saline sodium citrate buffer): 1 Liter

		Final concentration
NaCl	175.3g	3 M
Sodium Citrate	88.2g	0.34 M

### Processing of PFGE agarose gel before transfer onto the nylon membrane

Perform all the steps in a glass tray. A slice of gel representing run area from 1–2 plugs was removed followed by Ethidium bromide staining for 10-15 minute. The agarose gel can be sliced from either side or from the middle with no effect on subsequent steps. Place the gel on UV transilluminator to determine proper fractionation on the agarose gel.

25. Depurinate the DNA in the gel by soaking the gel in depurination solution (0.25M HCl) for 15–20 minutes. Approximately 200 mL depurination solution is required.
26. Wash the gel with water for 5 minutes.
27. Denature the gel containing fractionated DNA in denaturing solution (0.5M NaOH + 1.5M NaCl) for 25 minutes. Approximately 200 mL denaturing solution is required.
28. Repeat the denaturation step with fresh denaturing solution.
29. Discard the denaturation solution and rinse the gel with double distilled (dd)H_2_O.
30. Neutralize the gel by adding 250 mL of neutralizing solution and gently shake for 15 minutes.
31. Discard the neutralization solution and repeat the neutralization with fresh neutralization solution.

### Transfer of DNA onto the nylon membrane for Southern blot

32. Discard the neutralization solution from the previous step and rinse the gel with ddH_2_O.
33. Rinse the gel with ddH_2_O one more time.
34. Add approximately 250 mL 20X SSPE and keep for shaking for 30 minutes.
35. Soak the following while gel is shaking in SSPE.
  a. 1 nylon transfer membrane (of equal size of the gel) in ddH_2_O.
  b. 2 thin blotting papers in 10X SSPE.
  c. 1 thick blotting paper in 5X SSPE.
36. Remove and invert the gel over the platform of the capillary transfer system as shown in the, Figure 2). Put paraffin sheets along the sides of gel so that buffer should pass only through the gel.

**Figure 2:**
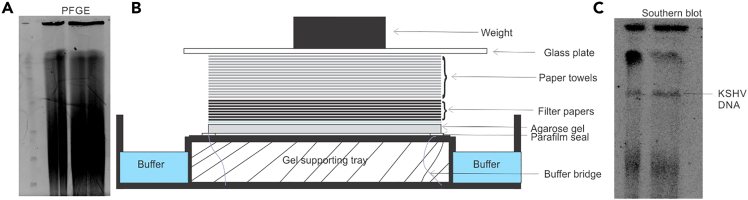
Representative images for pulse field electrophoresis gel (left), Schematic for setting up Southern blot (Middle) and a representative image of Southern blot showing region containing KSHV genome
37. Cover the gel with transfer membrane that was soaked in ddH_2_O. Remove all bubbles between the gel and membrane using a glass rod.
38. Cover with 2 thin blotting papers soaked in 10X SSPE.
39. Cover with 1 thick blotting paper soaked in 5X SSPE.
40. Cover with a stack of filter paper as shown in the Figure 2.
41. Put a glass plate on the top of assembly and a weight over the glass plate as shown in the schematic shown in Figure 2.
42. Let the transfer proceed for 12–16 hours.
43. Take out the membrane from the transfer assembly and crosslink the DNA by putting in UV chamber for 15 minutes at 1400V.

### Day 10: Preparation of radiolabeled probes for Southern blot

**Timing: 2–3 h**

Here we describe steps for preparation of radiolabeled hybridization probes which will be used in southern blot experiment.

44. Dilute 25 ng of template DNA (Not1 released fragment of pBS-PuroA plasmid, where a single copy of KSHV terminal repeat is cloned non-directionally in Not1 restriction site) and make up total volume up to 33 μL.
45. Denature the template DNA by boiling in a water bath for 5 minutes.
46. Quickly place the denatured DNA template on ice for 5 minutes followed by a brief centrifugation at 4°C.
47. Add the following components in the template containing tube in order.
  a. 5 μL Octa-deoxynucleotide in 10X labeling buffer.
  b. 6 μL dNTPs (2 μL each of dATP, dGTP, dTTP).
  c. 5 μL αP^32^dCTP (3000Ci/mmoles, 50 μCi).
48. Mix the components and incubate at 37°C for 1 hour.
49. Terminate the reaction by adding 5 μL 200mM EDTA, pH8.0.
50. Clean up the mixture from free nucleotides using a PCR clean up kit and elute the radiolabeled probe in 50 μL H_2_O.

***Note:*** All radioactive materials will need to be handled in designated area following institutional safety guidelines, using appropriate shielding, protective equipment, and waste disposal procedure to minimize exposure and ensure safe handling.

### Hybridization of radiolabeled probes to the DNA transferred onto the nylon membrane

51. Prepare the pre-hybridization buffer and Hybridization buffer as shown below.
  a. Pre-hybridization buffer.

**Table:** 

	For 10 mL	Final concentration
20X SSPE	3 mL	6X
50X Denhardt’s	2 mL	10X
20% SDS	0.5 mL	1%
ddH_2_O	4.5 mL	
Total	10 mL
  b. Hybridization buffer.

**Table:** 

	For 10 mL	Final concentration
20X SSPE	3 mL	6X
20% SDS	0.5 mL	1%
DdH_2_O	6.5 mL	
Total	10 mL

52. Put the membrane into the hybridization bottle and gently pour the pre-hybridization buffer to minimize bubbles. Perform the pre-hybridization for 10 hours to 12–16 hours at 49°C by gently rolling in incubator.
53. Change the pre-hybridization buffer with hybridization buffer containing radiolabeled probes and allow the hybridization for 10–12 hours to at 49°C, again rolling gently in incubator.

### Day 11: Washing of the membrane after hybridization

**Timing: 1 h**

Here we describe steps for washing of nylon membrane after hybridization with radiolabeled probes.

54. Prepare the washing buffers as given below.

**Table:** 6X Washing buffer

	For 30 mL	Final concentration
20X SSPE	9 mL	6X
20% SDS	0.3 mL	0.2%
ddH_2_O	20.7 mL

**Table:** High Stringency wash buffer

	For 30 mL	Final concentration
20X SSPE	1.5 mL	1X
20% SDS	0.3 mL	0.2%
ddH_2_O	28.2 mL

55. Remove the hybridization mix from the membrane and add 30 mL of 6X wash buffer. Wash the membrane for 15 minutes at 49°C.
56. Remove the 6X wash buffer and add 30 mL of high stringency wash buffer. Wash the membrane for 15 minutes at 49°C.
57. Wrap the membrane carefully in Saran Wrap without creasing and expose to a radioactivity sensitive plate. The plate can be scanned for radioactivity signals after 1-hour exposure. In case of low signal intensity, the exposure time can be extended further to 10–12 hours.

### Day 12: Gelase/Agarase digestion of the agarose gel and YOYO-1 staining

**Timing: 20–24 h**

Here we describe steps for digestion of agarose plugs by Gelase/Agarase followed by YOYO-1 staining of DNA.

58. Cut 3–4 slices of agarose 2–3 mm thick from the section that corresponds to the position of band on the Southern blot.
59. Wash the slices with TE8 buffer.
60. From each of the slice, cut agarose from one side of approximately 2 mm width. The agarose slices will be used for all the downstream experiment.
61. Equilibrate the agarose pieces in digestion buffer (TE8+ 100 mM NaCl+ 0.1% β-mercaptoethanol) for at least 1 hour at 4°C.
62. Transfer individual agarose pieces in a separate microcentrifuge and add 100–150 μL digestion buffer in each of the tube. Equilibrate the agarose pieces in the digestion buffer at 45°C for 5 minutes.
63. Melt the agarose at 68°C–72°C water bath for 15–20 minutes.
64. Equilibrate the melted agarose at 45°C for 2–3 minutes and add 2–3 μL gelase to digest agarose. Allow the gelase to complete digestion for at least 3–4 hours at 45°C. Alternatively, agarase can also be used as replacement of gelase. The recommended temperature for agarose digestion is 42°C.
65. Remove tube from the water bath and add 0.1 μL of YOYO-1 dye to each tube. Add β-mercaptoethanol to each tube to a final concentration of 5%. The DNA can be visible after 1–2 hours of staining. 12–16 hours staining generally results in better staining.
66. To view the DNA, put a coverslip on the prepared silanized slide and stretch the DNA from one of the edges of the coverslip. The stretching of the DNA is detailed in the next section. Visualize the DNA using a fluorescent microscope as described in the next step.

### Day 13: Stretching of the DNA on silanized slides and hybridization with KSHV-specific probes

**Timing: 1–1:30 h**

Here we describe steps for stretching of DNA on silanized slides followed by KSHV specific probes hybridization and immuno-detection of incorporated nucleotide analogs.

***Note:*** Three different fragments which represents different regions of KSHV genome with different sizes (6 KB, 10 KB and 15 KB) were used to prepare biotinylated probes, using nick translation method as per the reaction shown below. The KSHV genomic region for making the probes is selected such that directional alignment of individual KSHV DNA can be possible by visual differences in the length of consecutive signals.

Prepare KSHV specific probes by nick translation method using BioNick DNA Labeling System.

**Table:** 

DNA (6KB/10KB/15KB)	X μl (2 μg)	Final concentration
10 X dNTPs mix	5 μl	1X
10 X Enzyme mix	5 μl	1X
DdH_2_O	Up to 50 μl

67. Incubate the reaction mixture at 16°C for one hour. Run 5 μL of the nick translated product on agarose gel. The size of the fragments should be ideally in the range of 80–120 bp. The reaction time can be increased if fragments are higher in size or it can be decreased if more small fragments are observed.
68. Add 5 μL of stop buffer to terminate the reaction.
69. Remove the unincorporated nucleotides using a column and elute with 50 μL ddH_2_0.
70. Run 5 μL of the prepared probes on 1%–1.5% agarose gel to determine the average length of the prepared probes. Probes of excellent quality should be in the range of 80–150 bp.

**Prepare the hybridization buffer for biotinylated probes:** 100 μL of hybridization buffer is required for each slide to be hybridized. Prepare the hybridization buffer as per the table shown below.

**Table:** 

	For 1 mL	Final concentration
ddH_2_O	85 μL	
Formamide (purity>99%)	400 μL	40%
5M NaCl	200 μL	1 M
10% SDS	100 μL	1%
50% Dextran Sulfate	200 μL	10%
1M Tris pH 7.4	5 μL	5 mM
Salmon Sperm DNA (10 μg/μL)	10 μL	0.1 μg/μL

***Note:*** Salmon Sperm DNA is required as blocking reagent to prevent non-specific binding.

Prepare the following buffers for fixation and denaturation of DNA on silanized slides (Reagents for the use on the day of DNA stretching, Day-13).

71. Methanol + 0.1% β-mercaptoethanol.
72. Denaturation Buffer: 70% Ethanol + 0.1% β-mercaptoethanol+ 0.1 N NaOH.
73. Fixation buffer: Denaturation Buffer + 0.5% Glutaraldehyde.
74. Ethanol series: 70%, 95%, 100%.

### Steps for stretching DNA on silanized slides and hybridization

**Figure 3:**
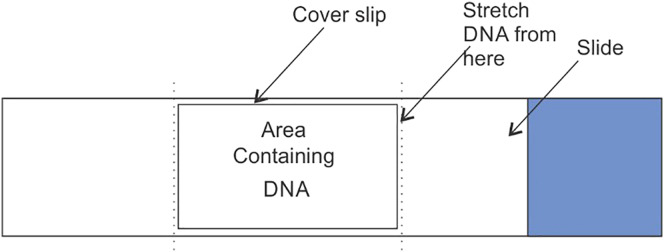
Schematic showing method to add YOYO-1 stained DNA on a silanized slide for stretching

75. Perform all the steps in Coplin jar filled with buffers as suggested in following steps.
76. Using a cut yellow tip (20–200 μL), spread 7 μL of the YOYO-1 stained DNA from one edge of the coverslip (22 X40) placed on the silanized slide as shown in Figures 3 and 4. For smaller size coverslip such as 22X22, a smaller volume (4 μL) of DNA is required.

**Figure 4:**
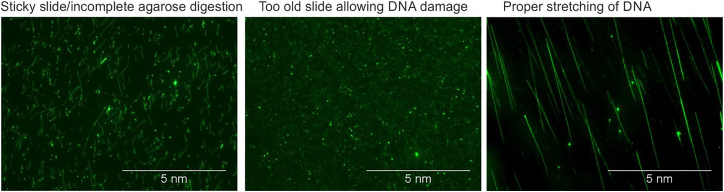
Representative images for YOYO-1 staining and stretching of DNA on silanized slides Sticky slide or incomplete agarose digestion (Left), Damaged DNA due to aged slide (Middle), Proper stretching of DNA on a good slide (Right).
77. Denature the DNA by dipping the slides in denaturation buffer for 15 minutes. Do not exceed denaturation for more than 20 minutes.
78. Freshly prepare fixation solution by adding glutaraldehyde in denaturation solution and fix the slides with fixation buffer for 5 minutes.
79. Wash the slides with 70%, 70%, 70%, 95%, 100% ethanol, 2–3 minutes each wash.
80. Replace the slides in methanol + 0.1% β-mercaptoethanol for 10 minutes.
81. Take out the slides from methanol + 0.1% β-mercaptoethanol solution, wipe the excess of buffer from sides of the slides using a Kimwipes paper and dry the slides at 20°C–25°C.
82. Assemble the coverslips on the slides as shown in the Figure 5 to create a hybridization chamber. Add 2-3 μL of hybridization buffer below the center of expected small coverslips positions. The buffer helps to adhere the coverslip with the slide. Similarly, put 2–3 μL of hybridization buffer above the small coverslips (22 x 22) where sides of long coverslip (22 x 40) will be expected to rest over small coverslip.

**Figure 5:**
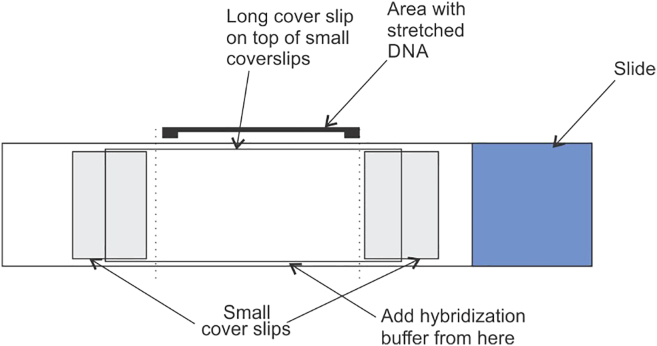
Representative image showing schematic for creating a hybridization chamber and adding KSHV-specific biotinylated probes
83. Prepare hybridization mix by adding biotinylated probes to the hybridization buffer. Make a separate tube for every slide. Take 94 μL of hybridization buffer and add 2 μL of each of the biotinylated probe.
84. Denature the hybridization mix containing biotinylated probes by heating at 95°C for 5 minutes.
85. Centrifuge the hybridization mix and pipette through one of the open sides of hybridization chamber.
86. Allow hybridization of the DNA and probes on slides by incubating the hybridization chamber (slides) 12–16 hours at 37°C in a humid box.

### Fluorescence *in situ* hybridization detection and immunostaining

Prepare the solutions for FISH detections and immunostaining. Make 20X stock for SSC and dilute in autoclaved water as needed according to various solution as listed below.

87. Solution 1: 4X SSC+ 40% Formamide (Set up 2 jars).
88. Solution 2: 2X SSC+ 1% SDS (Set up 2 jars).
89. Solution 3: 2X SSC+ 0.1% NP-40 (Set up 1 jars).
90. Solution 4: 4X SSC+ 0.1% NP-40 (Set up 4 jars).
91. Solution 5: 1X PBS+ 0.03% NP-40 (Set up 3 jars).
92. Solution 6 (blocking buffer): 3% BSA in 1X PBS.
93. Detection mix 1^st^ and 3^rd^: Dilute Neutr-Avidin Alexa 350 in blocking buffer (solution 6 above) in the ratio of 1:20. 20 μL will be required for each slide.
94. Detection mix 2^nd^: Dilute Biotinylated anti-Avidin in blocking buffer (solution 6 above) in the ratio of 1:20. 20 μL will be required for each slide.
95. Detection mix 4^th^: Dilute Biotinylated anti-Avidin (1:20), mouse anti-IdU (1:10) and rat anti-CldU (1:10) in blocking buffer (solution 6 above). 20 μL will be required for each slide.
96. Detection mix 5^th^: Dilute Neutr-Avidin Alexa 350 (1:20), Alexa 594-anti mouse (1:10) and Alexa 488-anti rat (1:10). 20 μL will be required for each slide.

### Stepwise procedure for detection

**Figure 6:**
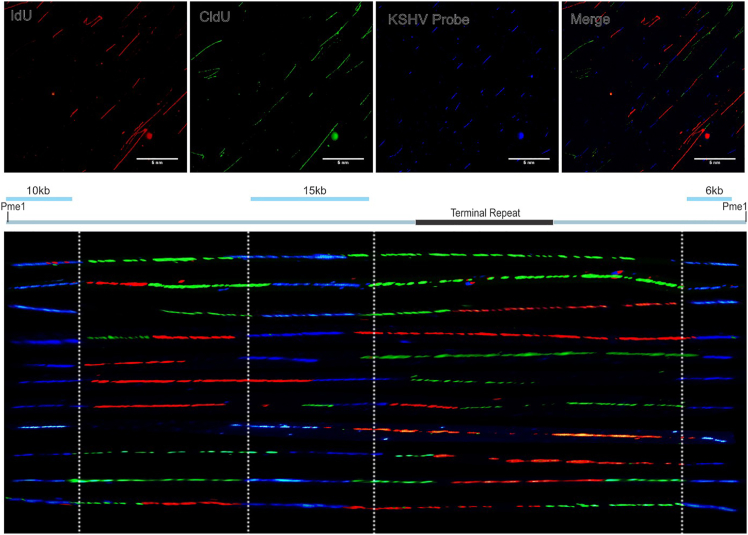
Representative image for immunostaining against individual nucleotide analogs and biotinylated probes (top) and merged enlarged image of KSHV-specific DNA showing KSHV-specific probed region and incorporated nucleotide analogs Merged images showing complete KSHV genome on various slides were manually aligned to show various KSHV genome (bottom).

97. Remove the coverslip using a razor blade. In a Coplin jar, rinse the slides in 2X SSC, 1% SDS.
98. Wash the slides in fresh 2X SSC, 1% SDS for 5 minutes.
99. Wash in 4XSSC, 40% Formamide (pre-equilibrated at 45°C) for 5 minutes. Move the slides up and down 2-3 times in between to remove excess of probes.
100. Rinse the slides in 2X SSC, 0.1% NP-40 at 20°C–25°C.
101. Wash in 4X SSC, 0.1% NP-40 at 20°C–25°C. Do this washing 4 times (5 minutes each) in a series in different Coplin jars.
102. Rinse in 1X PBS, 0.03% NP-40 at 20°C–25°C.
103. Using a dry Kimwipe, gently wipe off excess solution from back, side and front of the slides that do not have DNA molecules. Add 20 μL blocking buffer on the slides in the region of stretched DNA and cover with a coverslip. Incubate in a humid chamber at 20°C–25°C for 20–30 minutes.
104. Take off the coverslip using the edge of a razor blade and add 20 μL of detection mixture on each slide. Cover the slides with a new coverslip and incubate in a humid chamber at 20°C–25°C for 20 minutes.
105. Take off the coverslip by rinsing the slide in 1X PBS, 0.03% NP-40 and moving the slide up and down 3 times. Wipe excess buffer and add 20 μL detection mixture 2 on each slide. Cover the slide with a coverslip and incubate in a humid chamber at 20°C–25°C for 20 minutes.
106. Take off the coverslip by rinsing the slide in 1X PBS, 0.03% NP-40 and moving the slides up and down 3 times. Wipe out excess buffer and add 20 μL detection mixture 3 on each slide. Cover the slides with a coverslip and incubate in a humid chamber at 20°C–25°C for 20 minutes.
107. Take off the coverslip by rinsing slides in 1X PBS, 0.03% NP-40 and moving the slides up and down 3 times. Wipe out excess buffer and add 20 μL detection mixture 4 on each slide. Cover the slide with a coverslip and incubate in a humid chamber at 20°C–25°C for 1 hour.
108. Take off the coverslip by rinsing the slide through 1X PBS, 0.03% NP-40 and moving the slides up and down 3 times. Wipe out excess buffer and add 20 μL detection mixture 5 on each slide. Cover the slide with a coverslip and incubate in a humid chamber at 20°C–25°C for 1 hour.
109. Take off the coverslip by rinsing the slide through 1X PBS, 0.03% NP-40 and moving the slide up and down 3 times. Rinse the slides in a separate Coplin jar containing fresh 1X PBS, 0.03% NP-40 solution. Wipe out excess buffer from back and sides of slide that do not contain DNA.
110. Add 15 μL Antifade reagent on each slide and gently place a coverslip. Seal the coverslip with colorless nail polish and store the slides at 4°C for at least 1 hour before capturing the images on a fluorescent microscope (We capture the images on Olympus IX70 inverted fluorescence microscope). Alternatively, prolong gold can be also used for mounting purpose which do not require sealing with colorless nail polish.
111. Capture images (at 60X magnification) using individual color channel and merge the images using desired software such as ImageJ. A representative image (shown 6) which shows the individual images captured using different channels and merged are shown in Figure 6.

## Expected outcomes

The single molecule analysis of replicated DNA (SMARD) is a powerful technique which allows us to study DNA replication at the single-molecule level. The expected outcomes of SMARD include, but are not limited to, improved understanding of DNA replication dynamics such as the identification of replication origins, analysis of fork progression, and the influence of various regulatory factors on replication. The technique involves labeling newly synthesized DNA with nucleotide analogs (CldU and IdU) during the replication phase of the cell cycle. The labeled DNA is then isolated, stretched onto slides, and visualized using fluorescence microscopy, enabling precise tracking of replication fork location and movement. SMARD is particularly useful for studying replication in specific genomic regions or in the context of viral DNA, like those of Kaposi’s sarcoma associate herpesviruses (KSHV).

Through its high-resolution visualization capability, SMARD facilitates mapping of replication origins and termination regions while revealing replication fork dynamics, including progression and stalling events. It also provides insights into how environmental or experimental factors—such as hypoxia, oxidative stress, or viral infection—affect replication kinetics. Moreover, SMARD enables visualization of region-specific replication patterns that reflect the order and direction of fork movement, allowing distinction between early and late replication events through sequential labeling. The technique further supports investigation of viral replication processes in host cells, offering an approach to analyze how viral DNA integrates, replicates, or persists. Finally, SMARD helps identify genomic regions prone to replication-related mutagenesis or instability, thus contributing to a deeper understanding of genome maintenance and replication stress responses.

## Limitations

SMARD is technically demanding and requires meticulous handling of DNA fibers and labeling procedures. The process of isolating, stretching, and visualizing single DNA molecules can be challenging. SMARD is generally low-throughput because each molecule is analyzed individually. While it provides high-resolution data, collecting statistically significant results requires a substantial number of labeled molecules, which can be time-intensive. Additionally, the technique depends on the successful isolation of long, intact DNA molecules. The fragmentation or damage to the DNA during preparation can reduce the quality and length of analyzable DNA. SMARD is less effective for the analysis of small regions of interest or when replication of origins are close to each other. The concentration optimization is required for the nucleotide analogs (CldU and IdU) for labelling DNA of different cell types based on their replication inhibitory effects at higher concentration in sensitive cell types. Moreover, the technique is expensive and time-consuming. It also requires several reagents (e.g., nucleotide analogs and antibodies) and often uses advanced imaging systems. Further, In the case of integrated viral genomes, restriction sites near integration junctions may be absent or rearranged, requiring site mapping by long-read sequencing or engineering of alternative restriction sites via CRISPR-based approaches. Additionally, since SMARD captures replication intermediates, cells undergoing viral lytic replication may display heterogeneous patterns that should be interpreted with caution.

## Troubleshooting

### Problem 1

Sample preparation issues and contaminated or degraded DNA.

### Potential solution

While isolating and preparing agarose blocks and running the pulse field special care must be taken to ensure that there is no nuclease. Moreover, while running the Pulsed-field gel electrophoresis the temperature should be maintained at 16°C. The low temperature (16°C) prevents any possible heat mediated damage to agarose gel as the running of pulsed-field gel electrophoresis generates high heat. The low temperature is maintained by cooling module as shown in the Figure 1.

### Problem 2

Insufficient restriction endonuclease (PmeI) digestion leading to irregular separation.

### Potential solution

Make sure the protease digestion of cells is complete, otherwise the DNAs will not be released from the cell membrane. Moreover, after digestion deactivating the protease is important otherwise the subsequent restriction endonuclease (Pme1) treatment will be ineffective. Use of appropriate amount of restriction endonuclease (Pme1) is necessary to get properly digested DNA. Moreover, additional restriction endonuclease (Pme1) can be added next day to ensure restriction digestion. It is important to note that PmeI is generally insensitive to dam and dcm methylation but may be blocked by some combinations of overlapping CpG methylation. Therefore, digestion efficiency should be verified empirically for each sample.

### Problem 3

Inefficient or uneven labeling and photobleaching of fluorescent labels.

### Potential solution

Optimize the labeling conditions with primary antibodies and fluorescent dye (Alexa fluor) tagged Neutr-Avidin/secondary antibodies (e.g., time, temperature, and concentration of antibodies). The labelling of DNA with primary antibodies or alexa fluor conjugated Neutra-Avidin and secondary antibodies works best at 20°C–25°C. Please follow the incubation time with primary antibodies or Alexa fluor conjugated Neutra-Avidin and secondary antibodies strictly avoid under or over labelling. Use light sources (less energy) rather than leasers (high energy) for visualizing the stretched DNA as the high intensity leaser can break DNA strand.

### Problem 4

Optimal attachment of DNA to the Silane treated slides (see Figure 4).

### Potential solution

Use the slides after 12-24 hours of desiccation as the freshly prepared slides are very sticky and do not allow proper stretching. make sure DNA contains no traces of undigested agarose by ensuring proper agarose digestion with gelase/agarase. If the concentration of DNA is too high as seen after YOYO-1 staining, dilute the DNA before stretching again. Do not use Silane treated slides which are more than 5 days old.

### Problem 5

Microscopy/Imaging issues with poor signal-to-noise ratio.

### Potential solution

Check the focus and alignment of the microscope with some well-known working slides if available. Use appropriate filters and excitation power. Consider increasing the frame rate or exposure time to get better signal but balance it to avoid photodamage. Ensure that image misalignment is not caused by hardware limitations, especially when using non-motorized fluorescent microscopes. Misalignment or drift during image capture can cause poor focus. Additionally, high background fluorescence may reduce image clarity, this can often be minimized by optimizing washing steps or exposure settings.

### Problem 6

Low sensitivity in detecting individual DNA molecules and photodamage to DNA.

### Potential solution

Increase the concentration of fluorescent labels or use higher sensitivity detectors (e.g., EMCCD or sCMOS cameras). Use lower excitation powers and reduce exposure times to minimize photobleaching.

### Problem 7

Drift or misalignment in the optical system.

### Potential solution

As SMARD requires multichannel images, regular calibration of the microscope or other instruments used in the analysis is required. Otherwise aligning different channels after photo acquisition will be difficult.

### Problem 8

Probe preparation and hybridization not properly visible probe.

### Potential solution

Selecting the region of the genome to prepare probes is very important. There should not be too much secondary structures on the region and must be unique otherwise it will result in non-specific or no binding.

### Problem 9

Isolation of the KSHV genome from human genome if enough KSHV genome is not visible.

### Potential solution

Southern blot is performed to exactly locate the region in the agarose gel that contains the KSHV genome. Proper excision of the gel and dissolving the gel properly is important so that KSHV genome is more enriched, and they are stretched properly so that the KSHV specific probes can bind to it.

### Problem 10

Data Analysis and poor data resolution or difficulty identifying single molecules.

### Potential solution

Ensure to obtain adequate amount of data (large number of labelled KSHV DNA). For extracting information for replication origin and fork progression from SMARD images, alignment of several KSHV DNA molecules with differentially incorporated nucleotide analogs are required. Analysis of a large number of intact and labelled KSHV DNA molecules are required for extracting meaningful data. Typically, when concentration of DNA molecules while stretching on silanized slides are very high, overlapping molecules or molecules crossing one-another can be observed. Diluting the DNA sample can help minimize this problem.

## Resource availability

### Lead contact

Requests for resources and materials should be directed to the lead contact and corresponding author, Erle S. Robertson (erle@pennmedicine.upenn.edu).

### Technical contact

Technical questions on this protocol should be directed to and will be answered by the technical contacts, Erle S. Robertson (erle@pennmedicine.upenn.edu) and Rajnish Kumar Singh (rajsingh@pennmedicine.upenn.edu).

### Materials availability

All reagents are either commercially available or can be obtained from lead contact upon request and with a completed material transfer agreement.

### Data and code availability

No datasets or codes were generated in this study.

## Acknowledgments

This work was supported by the National Cancer Institute at the National Institutes of Health through the following awards to E.S.R.: P30-CA016520, R01-CA244074, R01-CA268998-01, and P01-CA281867.

## Author contributions

Conceptualization, R.K.S. and E.S.R.; investigation, R.K.S., D.B., and E.S.R.; writing – original draft, R.K.S., D.B., and E.S.R.; writing – review and editing, R.K.S., D.B., and E.S.R.; funding acquisition, E.S.R.; supervision, E.S.R.

## Declaration of interests

The authors declare no competing interests.
